# Teleconsultations for mental health: Recommendations from a Delphi panel

**DOI:** 10.1016/j.invent.2023.100660

**Published:** 2023-08-16

**Authors:** Valeria Manera, Claudia Partos, Olivier Beauchet, Michel Benoit, Benjamin Dupetit, Julia Elbaum, Roxane Fabre, Morgane Gindt, Auriane Gros, Rachid Guerchouche, Stefan Klöppel, Alexandra König, Annick Martin, Aurélie Mouton, Marie-Pierre Pancrazi, Antonios Politis, Gabriel Robert, Guillaume Sacco, Sabrina Sacconi, Kim Sawchuk, Fabio Solari, Lucille Thiebot, Pietro Davide Trimarchi, Radia Zeghari, Philippe Robert

**Affiliations:** a CobTeK laboratory, Université Côte d'Azur, Nice, France; b Association Innovation Alzheimer, Nice, France; c Department of Speech Therapy (Departement d'Orthophonie, DON), Université Côte d'Azur, Nice, France; d Budapest University of Technology and Economics, Budapest, Hungary; e Departments of Medicine and geriatrics, University of Montreal, Montreal, Quebec, Canada; f Research Centre of the Geriatric University Institute of Montreal, Montreal, Quebec, Canada; g Department of Medicine, Division of Geriatric Medicine, Sir Mortimer B. Davis Jewish General Hospital and Lady Davis Institute for Medical Research, McGill University, Montreal, Quebec, Canada; h Lee Kong Chian School of Medicine, Nanyang Technological University, Singapore; i Centre Hospitalier Universitaire de Nice, Service clinique gériatrique de soins ambulatoires, Centre Mémoire Ressources et Recherche, Université Côte d'Azur, Nice, France; j Nextfeeling, Cannes, France; k Côte d'Azur University, Nice University Hospital, Public Health Department, Nice, France; l Nice Pediatric Psychotrauma Center (NPPC), Child And Adolescent Psychiatry Department, Hôpitaux Pédiatriques Universitaires Lenval, Nice, France; m ki:elements GmbH, Saarbrücken, Germany; n University Hospital of Old Age Psychiatry, University of Bern, Bern, Switzerland; o Ville de Nice, Nice, France; p Centre Hopitalier de Bastia, Bastia, France; q Department of Psychiatry, National and Kapodistrian University of Athens, Eginition Hospital, Athens, Greece; r Academic Psychiatry Department, Guillaume Régnier Hospital, Rennes, France; s Empenn Inserm U1228, IRISA UMR 6074, Rennes University Hospital, France; t Université Cote d'Azur, Peripheral Nervous System and Muscle Department, Nice University Hospital, Nice, France; u Department of Communication Studies, Concordia University, Montreal, QC, Canada; v Department of Informatics, Bioengineering, Robotics and Systems Engineering, University of Genoa, Italy; w Provence Alpes Agglomération, Digne-les-Bains, France; x IRCCS Fondazione Don Carlo Gnocchi, Milan, Italy; y Université Cote d'Azur, Adult Psychiatry Department, Nice University Hospital, Nice, France

**Keywords:** Teleconsultations, Mental health, Telepsychiatry, SWOT analysis, Recommendations

## Abstract

**Introduction:**

The use of teleconsultations for mental health has drastically increased since 2020 due to the Covid19 pandemic. In the present paper, we aimed to analyze the advantages and disadvantages of teleconsultations for mental health compared to face-to-face consultations, and to provide recommendations in this domain.

**Methods:**

The recommendations were gathered using a Delphi methodology. The expert panel (N = 21) included professionals from the health and ICT domains. They answered questions via two rounds of web surveys, and then discussed the results in a plenary meeting. Some of the questions were also shared with non-experts (N = 104).

**Results:**

Both the experts and the non-experts with teleconsultation experience reported a general satisfaction concerning teleconsultations. A SWOT analysis revealed several strengths and opportunities of teleconsultations for mental health, but also several weaknesses and threats. The experts provided a set of practical recommendations for the preparation and organization of teleconsultations for mental health.

**Discussion:**

Teleconsultations for mental health have the potential to allow access to care for patients in remote and isolated areas. Thus, their use will unlikely be discontinued after the end of the pandemic. In this context, we suggest that the collaboration among clinicians, researchers, and interface designers is crucial to improve usability and user experience for both clinicians and patients. The importance of teaching teleconsultation skills and informing the public on the features of teleconsultations (e.g., data privacy/security) is also highlighted.

## Highlights

• Teleconsultations for mental health have the potential to allow access to care for patients in remote and isolated areas.
• The collaboration of clinicians, researchers and interface designers is crucial to improve usability and user experience for both clinicians and patients.
• Teaching teleconsultation skills and to inform the public on the features of teleconsultations (e.g., data privacy/security) is crucial.

## 1 Introduction

Teleconsultations have been available for many years, including in the domain of mental health (Tyrrell et al., 2001; Ball et al., 1998). Also referred to as telepsychiatry, teleconsultations for mental health concern not only psychiatrists, but also psychologists, psychotherapists, speech therapists, personal trainers, coaches, and all the paramedical professionals working to improve mental health and psychological well-being. In the last decade, teleconsultations for mental health have been regarded as a promising solution to reduce regional disparities in access to care (König et al., 2021). Indeed, teleconsultations have the potential to provide wider access to care to people living in remote areas, without obliging patients to move to specialized centers for assessment and care, thus improving cost-time savings and quality of life (Naslund et al., 2022). Several telemedicine systems have been developed (Zeghari et al., 2022; Chessa et al., 2021; Jurkeviciute et al., 2020), and there is some research-based evidence on their usability and usefulness, reporting sometimes conflicting results (Chan et al., 2015; Hilty et al., 2002; Monnier et al., 2003; Sharma and Devan, 2021), with some attempts to generalize telemedicine to everyday clinical practice (Das et al., 2020; Math et al., 2021).

The COVID-19 emergency produced a drastic shift towards telemedicine, including teleconsultations for mental health (Omboni et al., 2022; O'brien and McNicholas, 2020; Li et al., 2021), and caused the healthcare systems to adapt rapidly to digital healthcare solutions. In this context, some preliminary guidelines and recommendations are starting to emerge (Sivakumar et al., 2020; Greenhalgh and Wherton, 2022), with as well as some analyses of the advantages and disadvantages of teleconsultations for mental health (Gude et al., 2021). Psychiatrists acknowledged several advantages of teleconsultation, including time-cost reduction, increased safety, and the ability to see the patient without the mask, which favors a more natural conversation and improves therapeutic alliance (Thirthalli et al., 2020). However, recent surveys revealed that psychiatrists pointed out several drawbacks, including: the fear of potentially dangerous events while dealing with a suicidal or a homicidal patient; the risk of missing out on comorbidities, due to the inability to perform physical and central nervous system examinations; legal/regulatory issues due, for instance, to consultation recording; patients' inability to use conferencing devices; reduced feelings of closeness or connection; and technical problems (Basavarajappa et al., 2022; Guinart et al., 2021). Both patients and clinicians expressed concerns about establishing rapport, privacy, safety, and technology limitations, which can contribute to slowing down adoption (Basavarajappa et al., 2022). Surveys conducted on patients revealed general satisfaction with the quality of care in telepsychiatry, mainly regarding the perception of health care, but lower satisfaction with the doctor-patient relationship (Torales et al., 2023) and more difficulties establishing a therapeutic alliance, aggravated by the lack of physical proximity and nonverbal clues (Frittgen and Haltaufderheide, 2022). Taken together, these results suggest that “although telepsychiatry service is convenient for patients, the many barriers from clinicians' perspectives are concerning, because they serve as gatekeepers for implementation and sustainability of telepsychiatry services” (Cowan et al., 2019).

The objective of the present paper is to provide recommendations and operational guidelines, and to perform a SWOT analysis (Strengths, Weaknesses Opportunities, and Threats) on the use of teleconsultation for mental health. Despite the flourishing of recent works in this domain, we believe that the perspectives advanced in the present paper are relevant for several reasons. First, we followed a Delphi methodology, which is well established to collect the opinion of experts and reach a consensus on best practices in the healthcare domain (de Meyrick, 2003). Second, the expert panel is international, with representatives of countries with different social security systems (e.g., France, Canada, Greece, Italy, Hungary, Germany), which allow us to provide guidelines with international relevance. Third, the expert panel included psychiatrists, but also psychologists, speech therapists, and designers of telemedicine tools, thus including a wider range of professionals compared to previous recommendations, mainly based on the point of view of psychiatrists. Finally, we compared the opinion of the experts and of non-experts (potential patients), allowing the experts to reflect on barriers perceived by patients, and on ways to overcome them.

## 2 Methods

The expert panel included 21 professionals, 18 from the health domain (researchers and healthcare professionals, including six psychiatrists, two geriatricians, two neurologists, six psychologists/neuropsychologists, and two speech therapists), two from the ICT domain (researchers and engineers), and one coach/trainer. The experts were selected because they were partners of international research projects focused on teleconsultations for mental health, including the European projects Interreg-Alcotra “CLIP - E-santé-Silver Economy” and “PRO-SOL Senior”, and the EIT Digital Innovation Activity “ELEMENT”. Following a Delphi methodology (Linstone and Turoff, 1975), the recommendations were developed in a four-step process: after a literature review, the experts were asked to respond to questions in two rounds of web surveys. After each round, the facilitators (VM and PR) provided a summary of the experts' anonymous responses and encouraged the experts to comment on the results, with a focus on the points of disagreement among the experts. The experts were also asked to suggest further questions for the next round. The results were discussed in a final consensus meeting (as detailed below). All 21 experts participated in the two web surveys and the final consensus meeting. Some of the questions employed in the web surveys were also shared across non-experts, reached through existing mailing lists in France. The non-experts included students in the healthcare domain (e.g., speech therapy, psychiatry, psychology) at the Université Cote d'Azur in Nice, and people interested in research projects in the domain of healthcare, through the mailing lists established in the context of the Interreg-Alcotra project “E-santé Silver Economy” and from the Innovation Alzheimer Association in Nice. People were asked if they had previous experience with teleconsultations, but this was not employed as an inclusion criterion, as one of the purposes of the survey was to compare the opinions of people with and without direct teleconsultation experience. Responses were obtained from 104 volunteers (82 females and 22 males; 47 aged from 18 to 30 years, 21 between 31 and 50 years, and 36 between 51 and 90 years). In terms of socio-professional category, 40,4 % (N = 42) were students, 26 % (N = 27) were retired, 21,2 % (N = 22) were working in the health domain, 5,8 % (N = 6) in the education domain (teachers or researchers), and 6,7 % (N = 7) in other domains.

The final recommendations were drafted by the facilitators based on the results of the two surveys and the final consensus meeting, and were shared with all the experts for revision and approval.

### 2.1 Web-surveys

#### 2.1.1 Expert group

The experts were asked to answer questions via web surveys in two rounds (between February and April 2022) using Google Forms. Questions were based on a literature review performed by three of the authors (VM, PR, and CP). The question format was adapted from previous studies (Manera et al., 2017; Manera et al., 2022). Questions included rating questions, yes-no questions, multiple-choice questions, and open-ended questions. Rating questions employed a 5-point Likert scale (1 = Not important/pertinent at all; 2 = Not very important/pertinent; 3 = Important/Pertinent; 4 = Very important/pertinent; 5 = Extremely important/pertinent). After each rating question, participants could provide written comments. The open-ended questions for the experts in Delphi 1 round included providing a list of three advantages and three disadvantages of teleconsultations for mental health compared to face-to-face consultations. The expert responses were employed to guide a SWOT (Strengths, Weaknesses, Opportunities, and Threats) analysis of the use of teleconsultations for mental health. The two web surveys can be found in the Supplementary materials.

#### 2.1.2 General public

The web survey that circulated among the non-experts included a selection of the questions asked to the experts, such as questions on their experience with teleconsultations and the attitude of the clinician. The survey was circulated between March and April 2022.

### 2.2 Final consensus meeting

The results of the two web surveys and the open discussion points were revised by the task force during a hybrid plenary meeting held on April 28th, 2022, in Nice (France). 8 experts were physically present in Nice, while 13 were connected remotely. During the consensus meeting, the facilitators (VM and PR) presented the results of the two surveys combined, asking the experts to comment again and (eventually) revise their earlier answers considering the comments of other members of the panel. They also presented the results of the survey addressed to the non-experts. The facilitator took notes of all the discussion points and the full meeting was audio-recorded for transcription purposes.

### 2.3 Data analysis

The results of the yes-no questions and the multiple-choice questions were presented using the number and percentage of responses obtained for each alternative. Chi2 tests were employed to compare the percentage of responses among different groups. For the rating questions, we employed medians and the first and third quartile for descriptive analyses, and independent-sample *t*-tests for group comparisons. The analyses were performed using R-4.0.3.

## 3 Results

### 3.1 Expert group

#### 3.1.1 General questions on the use of teleconference systems for clinical vs. professional use

Results are reported in the Appendix (Supplementary Table 1) and therefore will not be discussed in detail here. In summary, most of the experts acknowledged that there is a difference in the tools, systems, and conditions of use of teleconferences between clinical consultations and professional meetings. Specifically, teleconsultations for mental health are mainly performed in a dedicated space, on a laptop/PC, and using dedicated software that complies with data security/privacy rules.

#### 3.1.2 Questions specific to mental-health teleconsultations

**Fig. 1:**
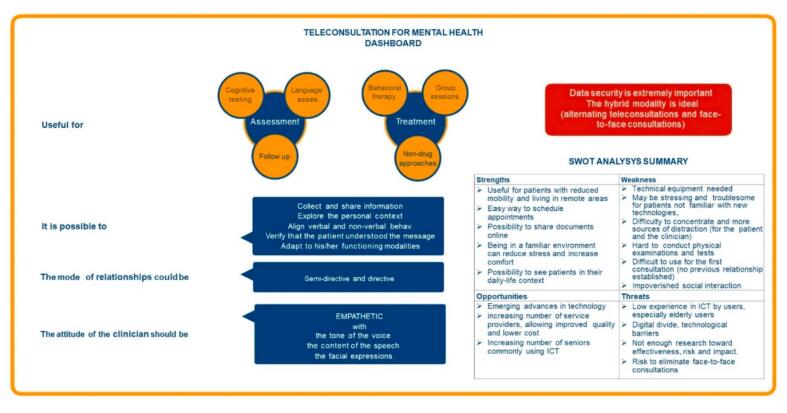
Teleconsultations for mental health dashboard. Based on the expert panel surveys, these guidelines are based on the “very important” and “extremely important” responses, and on the response options that reached at least 95 of agreement. The complete SWOT analysis is reported on Table 3.

Results are reported in Table 1, and the most important findings are summarized in Fig. 1. Concerning the content of mental-health consultations (Q1), the experts rated teleconsultations as ‘very pertinent’ for both assessment and treatment. Specifically, in terms of assessment (Q1a), the experts suggested that teleconsultations are ‘completely pertinent’ for follow-up consultations, and ‘very pertinent’ for cognitive testing and assessment of speech and language. They rated teleconsultations as ‘pertinent’ for the assessment of behavioral symptoms, physical function, and for first consultations. As specified in the open comments, this is mainly because it is more difficult to establish a therapeutic alliance in teleconsultations, which are usually shorter and provide limited access to non-verbal communication. In terms of treatment (Q1b), the experts rated it as ‘very pertinent’ to use teleconsultations for behavioral therapy, group sessions, and non-pharmacological approaches, and as ‘pertinent’ for physical training.

**Table 1:** Questions for the experts concerning teleconsultations for mental health (21 experts).

1. Telemedicine is useful for consultations devoted to	Median (Q1; Q3)
1a. Assessment	4 (3; 5)
First assessment	3 (2; 4)
Follow-up assessment	5 (4; 5)
Cognitive testing	4 (3; 4)
Physical function testing	3 (2; 3)
Assessment of behavioral symptoms	3 (2; 3)
Assessment of language	4 (3; 5)
1b. Treatment	4 (3; 5)
Behavioral therapy	4 (3; 4)
Physical training	3 (2; 3)
Group sessions	4 (3; 5)
Non-pharmacological approaches	4 (4; 5)
2. Is data security an important factor in the choice of the telemedicine system to use?	5 (4; 5)
3. How often should a mental-health teleconsultation be performed compared to a face-to-face consultation?	N (%)
Less frequently	2 (10 %)
At the same frequency	13 (62 %)
More frequently	6 (29 %)
4. Do you agree that a teleconsultation can be	
Directive	20 (95 %)
Semi-directive	21 (100 %)
Non-directive	16 (76 %)
5a. During a mental-health teleconsultation what should be the attitude of the clinician?	
Empathetic	20 (95 %)
Neutral	7 (33 %)
Inquisitory	5 (24 %)
5b. Do you agree that the empathy expressed is equivalent during a tele-consultation and a face-to-face consultation?	12 (57 %)
5c. During a teleconsultation empathy is expressed by clinicians through	Mean (IQR)
The number of words	3 (2; 4)
The content of the speech	4 (4; 4)
The tone of the voice	4 (4; 4)
The facial expressions	4 (3; 4)
5d. During a teleconsultation, it is possible to	
Explore the patient's personal context	5 (4; 5)
Adapt to his/her functioning modality	4 (4; 4)
Collect and share information	5 (4; 5)
Align verbal and non-verbal behavior	4 (3; 4)
Verify that the patient correctly understood the message	4 (4; 5)
6. Is it interesting to work in a hybrid way, alternating teleconsultations and face-to-face consultations?	5 (4; 5)

1 = Not important/pertinent at all/completely disagree; 2 = Not very important/pertinent/disagree; 3 = Important/Pertinent/Neutral; 4 = Very important/pertinent/agree; 5 = Extremely important/pertinent/completely agree.

Concerning the selection of the telemedicine system (Q2), they acknowledged that data security is an ‘extremely important’ factor to consider. In terms of frequency (Q3), the majority of the experts suggested that teleconsultations should be proposed at the same frequency as face-to-face teleconsultations. When asked about the format/style of the consultation (Q4), all the experts agreed that teleconsultations can be semi-directive but can also employ directive and non-directive formats.

The attitude of the clinician (Q5a) should be empathetic for most of the experts. When asked if the empathy expressed by clinicians during teleconsultations is the same as during face-to-face interactions (Q5b), only 67 % agreed. While they ‘agreed’ (Q5c) that during teleconsultations empathy can be expressed through the tone of the voice, facial expressions, and the content of the speech, in the comments they specified that there is a limited access to non-verbal behavior (e.g., body posture), and it is hard to make eye-contact, and impossible to have physical proximity/contact and movement synchronization, which are all important aspects to establish an empathetic relationship.

Teleconsultations were rated as ‘completely pertinent’ (Q5d) for anamnestic purposes, and to collect and share information. They are considered as ‘pertinent’ to verify that the patient understood the message delivered, to adapt to his/her functioning modality, and to align verbal and (facial) non-verbal behaviors. Finally, the experts rated it as ‘extremely interesting’ (Q6) to work in a hybrid way, alternating teleconsultations and face-to-face consultations.

### 3.2 Non-experts

As reported in Table 2, 54 % of the responders in the non-expert group declared (Q1) to have participated in a teleconsultation before. Those who had previous experience of teleconsultations, reported (Q1a) a median satisfaction concerning this experience of 4 out of 5 (the same as the experts). 46 % consulted a general practitioner, 38 % consulted a medical specialist, and 17 % another category of clinician (e.g., speech therapist, psychologist; Q1b). 81 % of them declared that the clinician, in their view, was as empathetic as during a face-to-face consultation (Q1c).

**Table 2:** Questions for the non-experts (N = 104) and comparisons with the experts (N = 21).

	Experts (N = 21)	General public (N = 104)
	N yes (%)	N yes (%)
1. Did you ever participate in a clinical teleconsultation?	18 (86 %)	56 (54 %)
	Median (IQR)	Median (IQR)
1a. If so, could you rate your global satisfaction?	4 (4;4)	4 (3;4)
1b. What practitioner did you consult?		N (%)
General practitioner		22 (46 %)
Specialist		18 (38 %)
Paramedical practitioner		8 (17 %)
1c. Do you agree that the clinician was as empathetic as during a face-to-face consultation?		39 (81 %)
		Median (IQR)
2a. Would you accept to talk about your personal psychological problems during a teleconsultation?		3 (2;4)
		N yes (%)
2b. Would you prefer to talk about your personal psychological problems during a teleconsultation compared to a face-to-face consultation?		7 (7 %)
3. Is doing a teleconsultation while you are at your place uncomfortable/embarrassing?		22 (21 %)
4. Do you think that the clinician during a teleconsultation is as attentive to you as during a face-to-face consultation?		55 (53 %)
5. Would you accept that your teleconsultation is recorder for your medical files?		57 (55 %)
6. Would you allow a member of the family to take part of the interview?	18 (86 %)	67 (64 %)
7. Telemedicine is useful for consultations devoted to	Median (IQR)	Median (IQR)
Assessment	4 (3;5)	2 (1;4)
Treatment	4 (3;5)	3 (2;4)
8a. Compared to the results of cognitive tests, do you consider the speech and the speech/discourse	N (%)	N (%)
Less informative	0 (0 %)	16 (15 %)
As informative	18 (86 %)	70 (67 %)
More informative	3 (14 %)	18 (17 %)
8b. Compared to the results of biological tests, do you consider the speech and the discourse of the patient		
Less informative	2 (10 %)	19 (18 %)
As informative	18 (86 %)	71 (68 %)
More informative	1 (5 %)	14 (14 %)

When participants were asked if they would feel comfortable talking about their personal psychological problems during a teleconsultation (Q2a), the median agreement was only 3 out of 5, and only 7 % of the respondents declared (Q2b) that they would prefer to talk about their psychological problems in a teleconsultation compared to a face-to-face consultation. The median rating was slightly higher for people that had previous experience with teleconsultation (3.5) compared to those without experience (3.0).

21 % of the responders agreed that doing a teleconsultation at home can be uncomfortable/embarrassing (Q3). There was a significantly higher proportion of persons that responded ‘yes’ among those without previous experience of teleconsultations (30 %) compared to those with previous experience (10 %, p = 0.013). In the same way, only 53 % of the respondents reported that, in their view, the clinician during a teleconsultation is as attentive to you as during a face-to-face consultation (Q4). However, the proportion of people that responded ‘yes’ was significantly higher for those with no teleconsultation experience (69 %) compared to those with experience (39 %, p = 0.003).

Only 55 % of the non-experts declared that they would agree that their teleconsultations are recorded and saved in their medical records, with no significant difference between those with and without teleconsultation experience (p = 0.144).

Concerning the presence of a family member during a teleconsultation (Q6), 64 % of participants in the public agreed that they would allow someone in the family to participate in the consultation, vs. 86 % of the experts (p = 0.056). When asked whether teleconsultations are pertinent for assessment and treatment purposes (Q7), the non-experts mainly disagreed on the interest of teleconsultations for assessment (median score = 2 out of 5, vs. a median score of 4 for the experts), while they rated the interest of using teleconsultations for treatment purposes a 3 out of 5.

For most of the experts and the public, what the patient says (speech and discourse) is ‘as important as’ the results of cognitive testing (Q8a; 86 % and 69 %, respectively, p = 0.142) and ‘as important as’ the results of biological tests (Q8b; 86 % and 68 %, respectively, p = 0.345), confirming that the information that can be most easily collected in teleconsultations is extremely valuable for assessment purposes.

## 4 Discussion

Drastically accelerated by the COVID-19 health crisis, teleconsultations are now widely employed in the clinical practice and are expected to play an important role in the future of medical care (Omboni et al., 2022). Consultations for mental health could represent one of the areas of wider telemedicine adoption, as they are mainly based on verbal exchanges (Torales et al., 2023). In the present paper, we collected the opinion of experts in the field and of non-experts to understand the satisfaction as well as the perceived barriers and limitations of telemedicine for mental health today, after two years of consistent use of telemedicine systems due to the Covid-19 pandemic (O'brien and McNicholas, 2020). We then provided recommendations to improve the technology and its use, to increase acceptability and facilitate wider adoption.

### 4.1 The view of the public

The results of the survey addressed to non-experts revealed that more than half of people had previous teleconsultation experience. The level of satisfaction of those with experience was quite high (4 out 5), converging with the level of satisfaction reported by the experts (4 out 5), and more than 80 % reported that the clinician was as empathetic as during a face-to-face consultation (see also (Torales et al., 2023)). Despite these encouraging results, some potential obstacles to mental health teleconsultations were also identified. Specifically, as a group, participants did not completely agree on the possibility to talk about their personal psychological problems during a teleconsultation. Also, while they agreed with the experts on the interest of using teleconsultation for training purposes, they reported being more skeptical on the possibility to use teleconsultations for assessment. This highlights the importance of research to understand the exact reasons for this resistance, for instance using qualitative interviews. Educational campaigns for the public may also improve acceptability. In addition, positive experiences may contribute modifying the patients' opinions. For instance, the fears that the clinician is less attentive during a teleconsultation and that doing a teleconsultation at home is embarrassing were significantly higher for people without teleconsultation experience. Another problem that emerged was the low confidence in data privacy/security, suggesting the importance of clarifying the privacy rules.

### 4.2 SWOT analysis on the use of teleconsultations for mental health

Several recommendations for telemedicine consultations were previously drafted in different domains (e.g., König et al., 2021; Omboni et al., 2022; Kaundinya and Agrawal, 2022). The experts in our panel were asked to focus specifically on mental health teleconsultations, and to list their advantages and disadvantages compared to face to face consultations. Their responses were used to formulate a SWOT (Strengths, Weaknesses, Opportunities and Threats) analysis (see Table 3 for a complete SWOT analysis, and Fig. 1 for a summary). Most of the features highlighted by the experts are valid for teleconsultations in general and were previously reported by other recommendation papers (e.g., König et al., 2021; Omboni et al., 2022; Manera et al., 2022; Kaundinya and Agrawal, 2022). We highlighted in bold in Table 3 the features that are more specific to mental health teleconsultations.

**Table 3:** Strengths, weakness, opportunities, threats (SWOT) analysis of teleconsultations for mental health. Most of the items are valid for all types of teleconsultations, while the elements in bold are especially important for teleconsultations for mental health.

Strengths	Weakness
- Useful for patients with reduced mobility - Useful for patients living in remote areas - Reduced travels (cost/time effectiveness, increased safety) - Increased health safety (e.g., no risk of virus contamination) - Can promote the maintenance of the therapeutic alliance through more frequent ad timely appointments (easier and faster to schedule) - The consultation is less time-consuming (for the patient and the clinician) - Useful for treatment/intervention monitoring and follow-up - Possibility to schedule appointments even during vacations, professional travel, or unrelated medical illness (e.g., Covid-19). - It can be performed outside a medical facility (e.g., a private professional does not need to rent an office) - Can allow to easily consult specialists outside the region - Possibility to share documents online (medical prescriptions, results of medical tests, etc.) - Possibility to see patients without masks - **Facilitate the involvement of the caregivers (important for patients that starts to lose their autonomy)** - **Can facilitate contacts with the clinical and paramedical team for patients leaving in nursing homes** - **Being in a familiar environment (e.g., at home) can reduce stress and increase comfort** - **Possibility to see patients in their daily-life context (important for the assessment of autonomy in activities of daily living)** - **Opportunity to influence daily activities in a therapeutic context** - **Possibility to easily record several “indirect” data (voice, gaze, movements, etc.)**	- Equipment (internet and/or device with camera and microphone) not available/reliable - May be stressing and troublesome for patients not familiar with new technologies, reducing treatment adherence - Technical problems may cause significant delay to connect or cause interruptions during the consultation - Additional time required at the beginning of the consultation to fix/optimize technical aspects (e.g., volume, camera position, etc.) - Difficulty to concentrate and more sources of distraction (for the patient and the clinician) if the consultation is not made in an adapted setting (other people in the room, doorbell, multitasking, etc.) - Hard to conduct physical examinations and tests - Hard to share medical records (e.g., IRM results performed outside the clinical facility) - **Privacy may become a concern (other people in the patients' house, or in the clinician's place)** - **May increase paranoid symptoms (who can see/spy the consultation?)** - **May be perceived as more intrusive by both the patient and the caregiver (visible objects, pets, etc.)** - **Reduced perceived intimacy and empathy, more impersonal** - **Difficult to use for the first consultation (no previous relationship established)** - **Impossible to touch the patient (for instance for welcome or reassurance purposes) and to react to distress situations** - **Impoverished social interaction** - **Non-verbal communication (other than facial expression) harder to assess, as only the upper body part is visible** - **Hard to detect informative details in the non-verbal behavior (e.g., trembling voice, tears, unusual gestures)** - **Difficulty to establish eye-contact (the clinician should look at the camera rather than the screen)** - **Not adapted for some interventions (such as EMDR protocols) and patients (advanced dementia)**


Opportunities	Threats
- Emerging advances in technology (telemedicine tools, internet coverage, etc.) - Good accessibility for users, also remotely (at home or in remote clinical facilities) - Digitalization of the results of most of the clinical assessments (e.g., MRI images shared with patients through files) - Use drastically accelerated by the Covid-19 health crisis - national social security systems and private insurance increased adoption, at no additional cost for the users - increasing number of service providers, allowing improved quality and lower cost - Governmental interest in promoting adoption to reduce costs and barriers in access to care in regions with limited access to specialized centers - Increasing number of seniors commonly using ICT - In case of positive experience, increased acceptability of ICT technologies	- Low experience in ICT by users, especially elderly users - Digital divide, technological/technical barriers (internet and/or device with camera and microphone not available in low-income countries/regions/families) - Reliability of remote assessment (e.g., neuropsychological tests) and treatment not yet fully validated - Not enough research evidence toward effectiveness, risk and impact. - Increase of isolation of non-connected individuals - Risk to eliminate face-to-face consultations to improve cost-time saving

#### 4.2.1 Strengths

Most of the strengths highlighted by the experts are not specific to mental health teleconsultations and are valid for telemedicine in different domains. These strengths include: the possibility of easily reaching people with reduced mobility and/or living in areas far from clinical facilities; the time-cost saving due to reduced travels, resulting also in increased security (e.g., accidents during commuting, risk of virus contamination); the possibility to schedule rapidly consultations, for instance to ensure treatment follow-up, which can also improve therapeutic alliance; this is also facilitated because teleconsultations are usually shorter; the possibility to perform teleconsultations virtually anywhere for the patient and the clinician, thus allowing to schedule appointments during vacations, travels, or unrelated medical conditions (e.g., Covid19 positive status). For private clinicians, this may reduce the necessity to rent a clinical office or at least change the classical standard rule for practitioner office. A room with a good connection and privacy may be enough if the clinician only practices using remote consultation, and it is also mandatory for those using hybrid format. Similarly, teleconsultations do allow contacting clinicians from virtually anywhere, if no specialist is available in the region, or to ask for a second opinion. Another strength is represented by the possibility of easily and rapidly sharing online documents (e.g., clinical prescriptions, results of tests).

Some of the strengths that were highlighted are especially important for mental health consultations. Teleconsultations allow for patients to be seen in a familiar, secure environment (at home). This can potentially reduce the stress/anxiety linked to the classical clinical setting, which do represent a common issue for patients with mental health issues. Teleconsultations facilitate the involvement of several family caregivers, and/or the professional caregivers (for example in nursing homes), thus allowing for more complete data collection on the patient's information, and to share treatment options/suggestions directly with the caregivers. The possibility to see patients in their daily-life context is also important for the assessment of autonomy in activities of daily living, and potentially offers the opportunity to influence daily activities in a therapeutic context (giving practical tips based on what the clinician can directly observe). Furthermore, teleconsultations allow recording, upon patient's explicit approval, several “indirect” data (voice, gaze, movements, etc.) that may be treated using machine learning or other tools to extract additional information helping diagnosis and treatment monitoring (e.g., (König et al., 2021)).

#### 4.2.2 Weaknesses

As for the strengths, most of the weaknesses highlighted by the experts are not specific to teleconsultations in the mental health domain. These include: the need of equipment and infrastructure allowing teleconsultations (e.g., device with camera and microphone, high-speed internet connection); the necessity to be able to employ this equipment, which may be challenging for elderly people and/or people not familiar with new technologies, potentially causing stress and lowering adherence to treatments proposed remotely. Technical problems may cause a significant delay in joining the appointment, a later time of the beginning of the consultation to fix/optimize technical aspects (e.g., volume, camera position, etc.), or cause interruptions during the consultation. The patient and the clinician can be more easily distracted, due to interruptions (e.g., someone entering the room, doorbell ringing) and multitasking (e.g., checking messages on the phone). In addition, it is hard to perform physical examinations, and to share/show medical records that are not available to the patient in a digitalized format (e.g., MRI scans). Privacy may become a concern if the consultation is performed in a non-private setting, as also suggested by (Omboni et al., 2022). This is especially important for mental health consultations, as patients must feel comfortable to share private and sensitive information. The sensation of reduced privacy may even increase paranoid symptoms (who can see/spy the consultation?). Also, consultation may be perceived as more intrusive (by both the patient and the caregiver) due to visible objects, pets, other persons, etc. On the other hand, some patients may experience a reduced perceived intimacy and empathy, considering the consultation more impersonal, representing an impoverished social interaction. During teleconsultations it is not possible to touch the patient, that is sometimes important for instance to welcome and reassure him/her, and to react to distress situations (tantrums, self-harm behaviors, etc.). Non-verbal communication is harder to assess, as only the upper body part is visible. It is harder to detect informative details in the non-verbal behavior (e.g., trembling voice, tears, unusual gestures), and even the details of the face are not always available, depending on the quality of the camera and connection. Importantly, it is almost impossible to establish eye-contact: the clinician should look at the camera rather than at the screen/patient to give the sensation that he/she is looking at the patient's eyes, which is unnatural. Finally, teleconsultations are considered as less adapted for some interventions (such as EMDR protocols).

#### 4.2.3 Opportunities

Emerging advances in technology (telemedicine tools, internet coverage, etc.) represent an opportunity to favor the adoption of telemedicine, improving accessibility for the users, also remotely (at home, or in remote clinical facilities). The digitalization of the results of most of the clinical assessments (e.g., MRI images shared with patients through files) may increase the potential for limiting face-to-face meetings, and make data sharing faster, even for patients that perform examinations outside the clinician's facility. Drastically accelerated by the Covid-19 health crisis, national social security systems and private insurances are increasing/pushing adoption, at no additional cost for the users. This is resulting in an increasing number of service providers, allowing improved quality and lower costs. Some tools are specifically devoted to mental health-related consultations, such as neuropsychological assessment (Zeghari et al., 2022). There is also an interest at the institutional/governmental level in promoting adoption, to reduce costs and barriers in access to care in regions with limited access to specialized centers. Finally, there is an increasing number of seniors commonly using ICT; in the case of positive experience with telemedicine, the result will be an increased acceptability of ICT technologies, promoting further use.

#### 4.2.4 Threats

Despite the increasing number of senior users, it is not uncommon to find elderly people with low experience in ICT, that are not motivated to learn, representing a barrier to the adoption of telemedicine for mental health. The equipment and infrastructure for teleconsultation are not available everywhere (e.g., in low-income regions) and to everyone, increasing the risk of digital divide. Another risk of switching to teleconsultations is that there is not enough research evidence concerning its effectiveness, risks, and impact. For instance, in the case of neuropsychological testing, the normative data collected during the face-to-face meeting are not necessarily adapted for internet-based testing, as the tests themselves require adaptations (e.g., using a mouse instead of a pen, or a screen or variable size instead of a standard A4 paper sheet). More research is needed to validate the digital test versions. Finally, the expert highlighted the risk that, driven by time-cost savings, the institutions will progressively eliminate face-to-face consultations for mental health practitioners. As highlighted before, face-to-face consultations are important sources of information, and the experts suggested the importance of a hybrid approach, combining video and face-to-face visits.

### 4.3 The expert recommendations

**Fig. 2:**
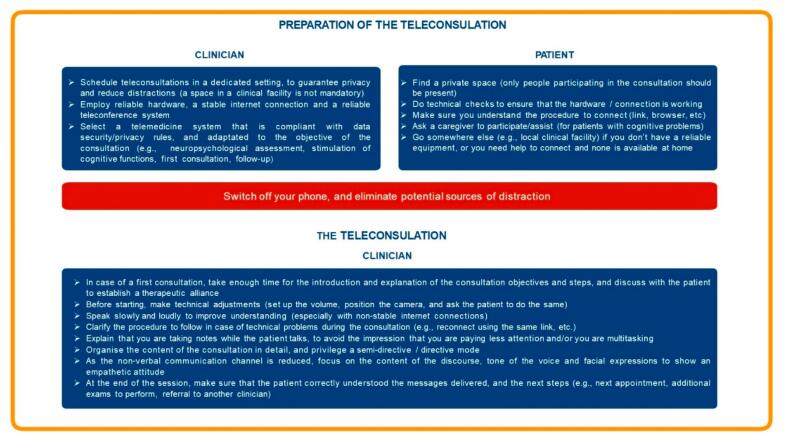
General recommendations and tips to conduct teleconsultation for mental health provided by the experts.

In Fig. 2 we summarized the practical recommendations and guidelines for teleconsultations for mental health provided by the experts, with a focus on the consultation preparation and delivery.

#### 4.3.1 Preparation of the teleconsultation

Converging with previous recommendations (Kaundinya and Agrawal, 2022; Furlepa et al., 2022), the experts suggested that it is crucial to schedule teleconsultations in a dedicated setting. While having a space in a clinical facility is not necessary for the clinician, the experts acknowledged the importance of having a private room where no other people can listen to the consultation, to guarantee privacy and reduce distractions. It is crucial to employ reliable hardware (e.g., PC/laptop with a good camera and microphone, and a fast and reliable internet connection) and a reliable teleconference system. While general-purpose systems are easier to employ and may be used as backup solutions in the case of temporary technical difficulties to connect to a dedicated system, it is important to select a telemedicine system that is compliant with data security/privacy rules, and which is approved by (or compliant with) the social security system. Based on the objective of the consultation (e.g., neuropsychological assessment, stimulation of cognitive functions, first consultation, follow-up) the clinician may employ different systems. It is important to avoid performing assessments with systems that are not designed for that purpose. For instance, performing neuropsychological assessments without a dedicated interface may provide biased results.

On the patient side, it is important to provide similar guidelines. For instance, the patient should be informed to try to find a private space, where only the people who participate in the consultation are present, and to do some technical checks beforehand to make sure that the hardware and connection are working correctly. It is important that the patient understands the procedure to connect (e.g., how to open the link in a web browser) and receives detailed information on how to do that (for instance, which web browser to use). Ideally, the patient should be allowed to perform a technical check (alone or connected with a technician) before the consultation to make sure that he/she knows how to connect. For elderly patients with cognitive problems, the experts advised suggesting the presence of a caregiver (as they do for a regular consultation). Setting up a technical facility where patients can go if they lack adequate equipment at home, and where there is a technician or a clinician able to help them to connect, is an ideal alternative solution.

Both the patient and the clinician should switch off their phones, and eliminate potential sources of distraction (e.g., switch off the TV, put a ‘no disturb’ sign on the door).

##### 4.3.1.1 Connection

After making pleasantries, it is important to make technical adjustments with the patient. This includes adjusting the volume so that the conversation is comfortable for both parties, asking the patient to position him/herself correctly in front of the camera, and verifying that the patient can see the clinician on full screen. The patient should be reminded to reduce the sources of distraction. In the case of a first consultation, it is important to verify the patient's identity, and to make sure that the patient is in a comfortable place (e.g., with privacy rules respected). Finally, it is important to explain the procedure in the case of technical problems (e.g., reconnect on the same link, wait for a phone call).

##### 4.3.1.2 Teleconsultation

The content/phases of the consultation should be well structured. The experts advised conducting semi-directive or directive consultations, while non-directive consultations may be harder to manage. In the case of a first consultation, it is important to take more time than during a face-to-face consultation for the general introduction and the explanation of the consultation objectives and steps, and to discuss with him/her in order to establish a therapeutic alliance and an empathetic relationship. As the non-verbal communication channel is reduced (body movement and posture), it is important to focus on the content of the discourse, tone of voice, and facial expressions to show an empathetic attitude. The clinician should provide explanations concerning the fact that he/she is taking notes while the patient talks, to avoid the impression that the clinician is paying less attention to them and/or he/she is multitasking. Due to potential technical difficulties (e.g., connection not 100 % stable), the clinician should pay attention to speaking slowly and loudly. At the end of the session, it is important to make sure that the patient correctly understood the messages delivered, and the next steps he/she should perform (e.g., next appointment, additional exams to perform, referral to another clinician).

### 4.4 Limitations

Despite its interest, we acknowledge some methodological limitations of the present work. First, not all the experts were clinicians (we also included two researchers in the engineering domain and one sport coach). These experts were included because they were part of projects based on telepsychiatry, and could bring a complementary, technical point of view in terms of telemedicine tools and technological barriers, or information on domains indirectly related to mental health (e.g., physical training). However, they could not respond to all the questions, thus reducing the number of responses for the clinical-related topics. Second, the non-experts were recruited only in France from existing mailing lists, which could represent a selection bias reducing the sample representativity.

## 5 Conclusions

The objective of the present paper was to analyze the advantages and disadvantages of teleconsultations on mental health (psychiatry, psychology, speech therapy) from the point of view of a panel of experts in the domain, and to provide recommendations for clinicians involved in mental health consultations, based on the experts' experience, and on the problems highlighted by non-experts (potential patients). Taken together, and converging with previous work (Gude et al., 2021; Torales et al., 2023), our results suggest that teleconsultations for mental health are well accepted by clinicians and patients. However, to further increase acceptance and usability, some additional work should be done to raise awareness among the patients on the teleconsultation rules and features (e.g., the clinician is as attentive as during a face-to-face consultation; the consultation is not impersonal). Similarly, clinicians should be trained on the specific features of teleconsultations, to avoid increasing patients' misconceptions (e.g., the importance of explaining that they are taking notes and not talking with someone else, and of telling the patient that their privacy is respected). As previously reported, performing teleconsultations for mental health raises new learning needs, and postgraduate training should integrate the teaching of specific skills required for the practice of effective mental health consultations (Omboni et al., 2022; Crawford et al., 2016). These skills include not only technical skills, such as correctly using telemedicine tools, but also soft skills, such as the capacity to form a therapeutic alliance, the flexibility in the structure of the interview, while being able to complete the full interview in the allotted time, and where to appropriately incorporate family members. For instance, at the Université Cote d'Azur, we recently created training modules for students in the clinical domain specifically devoted to teleconsultation (see Appendix). The designers of telemedicine systems could play a role in increasing acceptability, for instance designing interfaces that improve the impression of establishing eye contact with the patient while looking at the screen, and designing systems allowing to perform easily standard tests/tasks that require showing images or require the patient to respond non-verbally. Similarly, researchers should validate the use of these platforms compared to face-to-face testing, to reassure clinicians and patients on the comparability of the results obtained. The collaborative work of researchers, clinicians, and interface designers is thus crucial to further advance the field.

## Declaration of competing interest

The authors declare that they have no known competing financial interests or personal relationships that could have appeared to influence the work reported in this paper.

## Appendix A Supplementary data

Supplementary material Image 1
