# Supplementary material for: Teleconsultations for mental health: Recommendations from a Delphi panel
Source: Internet Interv. 2023 Aug 16;34:100660. doi: 10.1016/j.invent.2023.100660 (PMC10465930; doi:10.1016/j.invent.2023.100660)
Supplement: Supplementary file 1 — Supplementary material [file mmc1.docx]

**Appendix**

**Supplementary Table 1**. General questions concerning the experts’ use of teleconferences for professional vs clinical use

|  | Tele-consultations with patients (N=14*) | Professional meetings (N=21) |
| --- | --- | --- |
| 1. How often do you use tele-conference systems? | | |
| Never | 1 (7%) | 1 (5%) |
| Occasionally | 6 (43%) | 1 (5%) |
| Often | 2 (14%) | 1 (5%) |
| Frequently | 5 (36%) | 18 (86%) |
| 2. Is there a difference between tele-conference tools/systems (and their use) for professional meetings vs. tele-consultations with patients? (N=21) | | |
| Yes | - | 14 (67%) |
| No | - | 7 (33%) |
| 3. Which tele-conference system do you use for | | |
| Zoom | 5 (36%) | 20 (95%) |
| Teams | 2 (14%) | 18 (86%) |
| Skype | 0 (0%) | 8 (38%) |
| WhatsApp | 0 (0%) | 5 (24%) |
| Other commercial platform | 1 (7%) | 4 (19%) |
| Telemedicine commercial platform | 7 (50%) | 0 (0%) |
| Home-developed telemedicine platform | 5 (36%) | 1 (5%) |
| 4. Which kind of device do you use | | |
| Laptop | 9 (64%) | 21 (100%) |
| Smartphone | 1 (7%) | 15 (71%) |
| PC | 9 (64%) | 10 (48%) |
| Tablet | 1 (7%) | 3 (14%) |
| Other | 1 (7%) | 0 (0%) |
| 4. Where do you do | | |
| In the business office | 14 (100%) | 19 (90%) |
| At home, in a dedicated space | 5 (36%) | 16 (76%) |
| At home, in a common space | 1 (7%) | 9 (43%) |
| Other | 0 (0%) | 3 (14%) |

* Seven out of the 21 experts were not concerned directly by clinical consultations with patients (because they were mainly working on research-related or technical aspects of teleconsultations). The percentage of responses for questions concerning clinical consultations is then based on a total of 14 responses.

**Supplementary files**

**Delphi 1 Web-survey**

1. Did you ever participate in a clinical teleconsultation?

- Yes
- No

1. If so, could you rate your global satisfaction for this experience?

- 1. Not satisfied at all
- 2
- 3
- 4
- 5 Totally satisfied

1. How often do you use tele-conference systems for teleconsultations with patients?

- Never
- Occasionally
- Often
- Frequently
- Not concerned

1. How often do you use tele-conference systems for professional meetings?

- Never
- Occasionally
- Often
- Frequently
- Not concerned

1. Which tele-conference system/s do you use for teleconsultations with patients?

- Zoom
- Teams
- Skype
- WhatsApp
- Doctolib
- Telemedicine commercial platform
- Home-developed telemedicine platform
- Other commercial platform
- Other

1. Which tele-conference system do you use for professional meetings?

- Zoom
- Teams
- Skype
- WhatsApp
- Duo
- Other commercial platforms
- Other

1. Which kind of device do you use for teleconsultations with patients?

- Smartphone
- Laptop
- Pc
- Tablet
- Other

1. Which kind of device do you use for professional tele-meetings?

- Smartphone
- Laptop
- Pc
- Tablet
- Other

1. Is there a difference between tele-conference tools/systems (and their use) for professional meetings vs. tele-consultations with patients?

- Yes
- No
- Please comment

1. Where do you do your teleconsultations with patients?

- In the business office
- At home, in a dedicated space
- At home, in a common space
- Other

1. Where do you do your professional teleconference meetings?

- In the business office
- At home, in a dedicated space
- At home, in a common space
- Other

1. In your opinion, on a scale from 1 to 5 (1= Not at all, 5= Totally) telemedicine is useful for consultations devoted to

- Assessment
- Treatment
- Please comment

1. During a mental health teleconsultation what should be the attitude of the clinician? (Multiple choice)

- Neutral
- Empathetic
- Inquisitory
- Please comment

1. How often should a mental-health teleconsultation be performed compared to a face-to-face consultation?

- Less frequently
- At the same frequency
- More frequently
- Please comment

1. Can you list 3 advantages of mental health teleconsultation compared to a classic consultation?
2. Can you list 3 disadvantages of mental health teleconsultation compared to a classic consultation?

**Delphi 2 Web-survey**

1. Is it interesting to work in a hybrid way, alternating teleconsultations and face-to-face consultations?

- 1. Not at all
- 2
- 3
- 4
- 5 Totally
- Please comment

1. Is data security an important factor in the choice of the telemedicine system to use?

- 1. Not at all
- 2
- 3
- 4
- 5 Totally
- Please comment

1. On a scale from 1 to 5 (1= Not at all, 5= Totally) telemedicine is useful for consultations devoted to

- First assessment
- Follow-up assessment
- Cognitive testing
- Physical function testing
- Assessment of behavioral symptoms
- Assessment of language
- Behavioral therapy
- Physical training
- Group sessions
- Non-pharmacological approaches
- Please comment

1. Compared to the results of cognitive tests, do you consider the speech and the speech /discourse

- Less informative
- As informative
- More informative
- Please comment

1. Compared to the results of biological tests, do you consider the speech and the discourse of the patient

- Less informative
- As informative
- More informative
- Please comment

1. During Delphi 1, most responses indicated empathy as the most appropriate attitude for clinicians. Is empathy during a video-consultation equivalent to that of a face-to-face consultation?

- Yes
- Non
- Please comment

1. During a teleconsultation empathy is expressed by clinicians through (select all which applies)

- The number of words
- The content of the speech
- The tone of the voice
- The facial expressions
- Please comment

1. Do you agree that a teleconsultation can be (select all which applies)

- Directive
- Semi-directive
- Non-directive

*Definitions*
Directive interview: specific and numerous questions.
Semi-directive interview: general and fewer questions.
Non-directive interview: the patient is only invited to explain his/her problem, no questions.

- Please comment

1. During a teleconsultation, it is possible to

- Explore the patient’s personal context
- Adapt to his/her functioning modality
- Collect and share information
- Align verbal and non-verbal behavior
- Verify that the patient correctly understood the message
- Please comment

1. Would you allow a member of the patient's family to participate in part of the interview?

- Yes
- No
- Please comment

1. During Delphi 1, the following advantages concerning psychiatric visio-consultations were indicated. Please comment.

- List

1. During Delphi 1, the following disadvantages concerning psychiatric visio-consultations were indicated. Please comment.

- List

**TeachMod**

In medical psychology and psychiatry, the patient-caregiver relationship during a consultation is the keystone of an appropriate diagnosis and therapeutic success. This is particularly important for subjects with cognitive impairment. However, due to a lack of standardization, internship opportunities, and sometimes time, teaching these skills is sometimes difficult. TeachMod is an educational and interactive tool aimed at teaching some of the relational basics to use during a consultation.

TeachMod was developed with Unity3D (real-time 3D engine) by integrating two omnidirectional videos: one in traditional consultation (face to face), and the other, in **teleconsultation**. Each video includes 16 critical situations highlighted by the embedding of augmented reality elements with which the student can interact. A question is asked for each situation. The student must choose the most relevant answer. A summary of the questions and a score are displayed at the end of each video, so that the student identifies his level in relation to the acquisition of his skills.

TeachMod was used by 65 students from the Master 2 in Speech Therapy at the University of the Côte d'Azur. The presentation of TeachMod , the application in French and all the results of the survey are available on [www.innovation-alzheimer.fr/relation-soigne-soignant/](http://www.innovation-alzheimer.fr/relation-soigne-soignant/)

The full report is available on the following reference also available on the link:

*Mael Addoum, Yannick Bourquin, Quentin Bleuse, Auriane Gros, Jean Breaud, Marilou Serris, Philippe Robert. An Interactive Module for Learning and Evaluating the Basic Rules in Health Consultations. 2022 IEEE Conference on Games" https://ieee-cog.org/2022/*
